# Human *ACE2* Gene Replacement Mice Support SARS-CoV-2 Viral Replication and Nonlethal Disease Progression

**DOI:** 10.4049/immunohorizons.2400030

**Published:** 2024-09-17

**Authors:** Joshua M. Thiede, Jenna K. Dick, Nicholas N. Jarjour, Venkatramana D. Krishna, Lily Qian, Jules Sangala, Kellie Benzow, Kul Karanjeet, Shine Chin, Orion Rainwater, Maxim C.-J. Cheeran, Kristin A. Hogquist, Stephen C. Jameson, Geoffrey T. Hart, Tyler D. Bold, Michael D. Koob

**Affiliations:** * Center for Immunology, University of Minnesota Medical School, Minneapolis, MN; † Department of Medicine, University of Minnesota Medical School, Minneapolis, MN; ‡ Department of Laboratory Medicine and Pathology, University of Minnesota Medical School, Minneapolis, MN; § Department of Veterinary Population Medicine, University of Minnesota College of Veterinary Medicine, St. Paul, MN

## Abstract

Many mouse models of SARS-CoV-2 infection involve expression of the human ACE2 protein, the entry receptor for SARS-CoV-2 Spike protein, in mouse tissues. However, most of these models suffer from nonphysiological regulation of ACE2 expression, which can lead to atypically severe infections and aberrant sites of viral replication. In this report, we developed and characterized an *ACE2* gene replacement (*ACE2*-GR) mouse strain in which the mouse *Ace2* genomic locus was replaced by the entire human *ACE2* gene locus, and we investigated the ability of these animals to respond to SARS-CoV-2 infection. We show that *ACE2*-GR mice support SARS-CoV-2 viral replication, but, in stark contrast to the widely used K18-hACE2 transgenic model, this infection leads to a mild disease with no detectable involvement of the CNS. Thus, *ACE2*-GR mice provide a novel, to our knowledge, model to explore immune responses and long-term consequences of SARS-CoV-2 infection.

## Introduction

Infection with SARS-CoV-2, the causative agent of COVID-19, provokes varied disease states in humans, ranging from asymptomatic infection to severe respiratory disease and death. Animal models are critical to providing insights into mechanisms of infection and pathology, the efficacy of treatments and vaccines, and the ability to respond to related coronaviruses that are likely to emerge. Although models already exist to enhance our understanding of the pathology associated with acute, severe COVID-19, there is an urgent need to model other outcomes of SARS-CoV-2 infection (1).

Mice offer unique advantages for genetic manipulation of factors that contribute to viral control and disease in vivo. However, current mouse models for COVID-19 have substantial limitations, based largely on the way in which viral entry is modeled (2). Like SARS-CoV-1 (3), the Spike (S) protein on SARS-CoV-2 binds to human angiotensin-converting enzyme 2 (ACE2), permitting cell entry (4–6). The first strains of SARS-CoV-2 studied were unable to bind to mouse Ace2 protein, and, although the S protein on some subsequent variants of SARS-CoV-2 can access cells through Ace2 (6–8), it is not clear how well the affinity of these interactions and the regulation of binding compare with Spike protein engagement with human ACE2. Studies in which spontaneous variants of the SARS-CoV-2 S protein were selected for binding to mouse Ace2, through serial mouse infection (9–12), have the same limitations.

To focus on mouse models in which SARS-CoV-2 infects cells via human ACE2, transgenic mice expressing human ACE2 have been widely used, including models that had been developed for SARS-CoV-1 research. Other studies employed transient ACE2 expression in the lungs through intranasal infection of mice with recombinant adenovirus or adeno-associated virus vectors (2, 13). A recognized limitation of all these models (14) is the dysregulated expression pattern of exogenous ACE2. For example, a widely used transgenic model involves human *ACE2* cDNA under the control of the keratin 18 (K18) promoter (15) (*K18-hACE2*). These mice rapidly die following intranasal SARS-CoV-2 infection and show marked neurodissemination of the virus (16), which correlates with aberrant expression of the hACE2 transgene in the neuroepithelium and may contribute to the rapid death of infected *K18-hACE2* mice (17). Other transgenic lines involve expression of the full-length human *ACE2* cDNA under the control of the mouse *Ace2* promoter; although these animals were less vulnerable to lethal outcomes of SARS-CoV-2 infection (18, 19), it is unclear whether mouse *Ace2* regulatory elements adequately recapitulate the expression pattern of the human *ACE2* gene. Indeed, recent studies have shown that the expression pattern of human *ACE2* and mouse *Ace2* differ because of both upstream promoter and intragenic elements (20).

Studies using recombinant adenovirus and adeno-associated virus vectors encoding *ACE2* transgenes have focused on transduction of lung epithelial cells and cause strong but transient expression in those tissues, with minimal if any expression in other cell types or tissues (2). In contrast to these models, the human *ACE2* gene is expressed at high levels in tissues such as the kidney and intestines but low (often barely detectable) expression in the lung (21). Such temporary and aberrant hACE2 transgene expression patterns make these approaches of limited value in modeling physiological SARS-CoV-2 infection.

Finally, nearly all the current models for expressing human ACE2 in mice employ the full-length human *ACE2* cDNA, precluding alternative splicing. This is relevant because studies in humans indicate inflammatory cues can induce alternative splice forms of *ACE2* that may differ in their ability to serve as a receptor for SARS-CoV-2 (20, 22–25).

Therefore, we sought to characterize SARS-CoV-2 infection in a mouse strain in *ACE2* gene replacement (*ACE2*-GR) mice, in which both the human *ACE2* coding sequence and the flanking genomic regulatory elements replace the syntenic mouse *Ace2* locus. In these mice, the key gene regulatory elements upstream and downstream of the human *ACE2* coding sequence are present and permit expression of alternative spliced forms of *ACE2*. In addition to providing the appropriate genomic context for the human *ACE2* gene, removal of the entire mouse *Ace2* locus avoids potential artifacts due to coexpressing mouse and human ACE2 proteins in the same animals. We used mice with the *ACE2*-GR locus on the C57BL/6 background because this strain has many genetic and immunological tools to help study the immune response to SARS-CoV-2. We report in this study that these mice express human ACE2 in expected tissue sites and support efficient replication of SARS-CoV-2 following intranasal infection. However, unlike *K18-hACE2* transgenic mice, SARS-CoV-2 infection of *ACE2*-GR mice resembled an asymptomatic infection in which the virus did not cause lethality or discernible weight loss and did not spread to the brain. *ACE2*-GR mice reliably make an Ab response to SARS-CoV-2 infection, indicating that these animals may be useful to investigate immunological responses to SARS-CoV-2 infection in the context of more physiological human ACE2 expression patterns. Importantly, these *ACE2*-GR mice have already been deposited at The Jackson Laboratory (JAX 035800) and hence are accessible to the research community.

## Materials and Methods

### *ACE2*-GR mice

**FIGURE 1.**
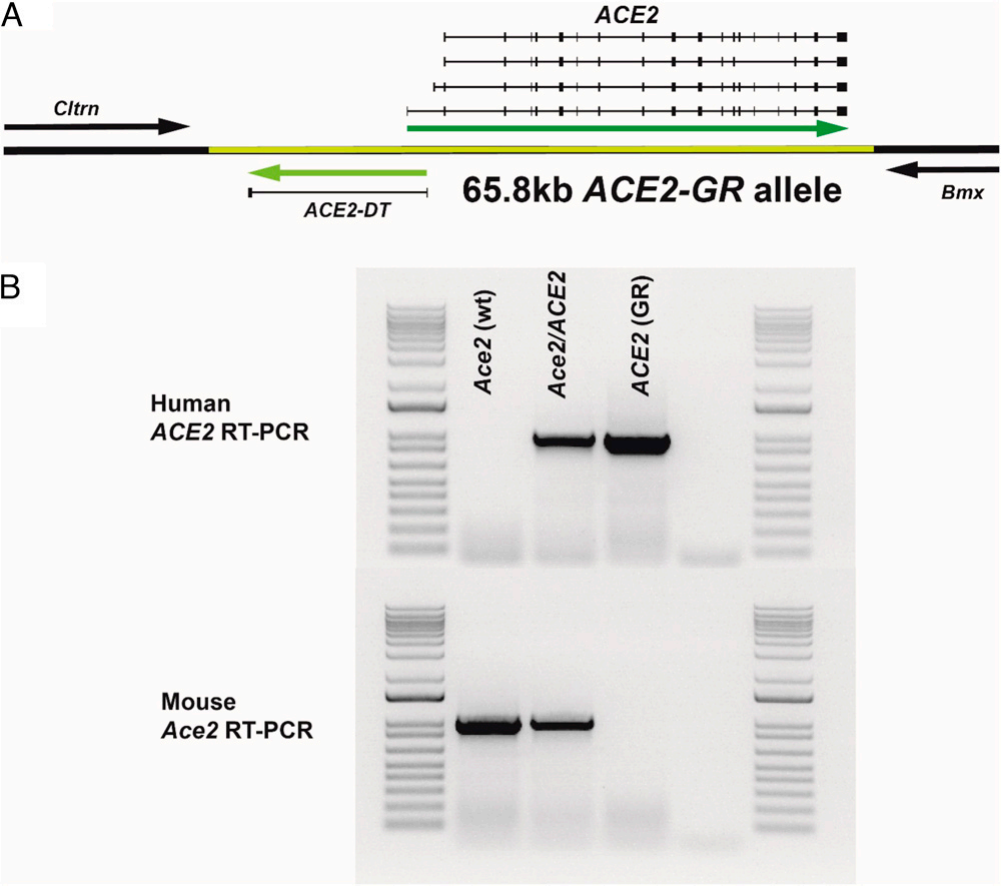
The full human *ACE2* gene replaces the mouse *Ace2* gene in *ACE2*-GR mice. (**A**) The segment of the mouse genome flanked by the *Cltrn* and *Bmx* genes (shown as black arrows) and encoding the mouse *Ace2* gene (71 kb) was precisely and completely replaced by the syntenic region of the human genome that includes the human *ACE2* gene (encoding human ACE2) and the long noncoding *ACE2*-*DT* (human sequences are shown in green). The exon structures of several *ACE2* alternative splice transcripts are shown. (**B**) Expression of *Ace2* (mouse) and *ACE2* (human) transcripts in kidneys of WT, *ACE2*-GR heterozygous, and *ACE2*-GR homozygous mice by species-specific RT-PCR.

In *ACE2*-GR mice, a 71-kb segment of the mouse genome encoding the *Ace2* gene and flanked on either side by the mouse genes *Cltrn* and *Bmx* is precisely replaced by the 65.8-kb syntenic region of the human genome (Fig. 1). The human GR region encodes all known *ACE2* promoter sequences and transcribed regions, as well as the *ACE2* divergent transcript (*ACE-DT*) long noncoding RNA. This human allele is maintained in a C57BL/6J background strain and was made available through donation to The Jackson Laboratory in August 2021 (JAX strain 035800).

### ACE2 mRNA expression

**FIGURE 2.**
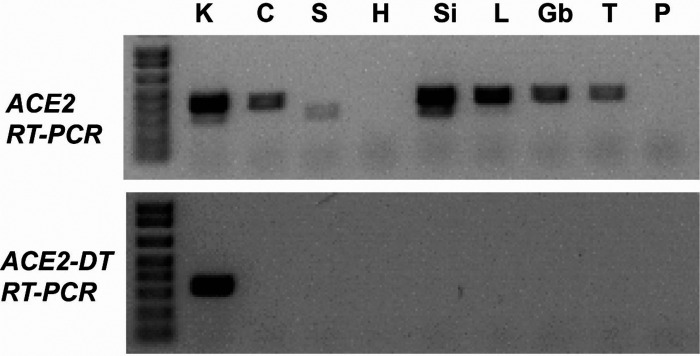
Human *ACE2* and *ACE2-DT* expression in *ACE2*-GR mice. The indicated tissues were harvested from *ACE2*-GR mice, and mRNA expression was evaluated by RT-PCR assays (as described in Materials and Methods) (C, colon; Gb, gallbladder; H, heart; K, kidney; L, liver; P, pancreas; S, stomach; Si, small intestine; T, testis).

Total RNA was isolated from mouse tissue using TRIzol (Thermo Fisher, 15596026). Tissue was homogenized (100 mg tissue/1 ml TRIzol), incubated for 5 min, and extracted with 200 µl chloroform, and then the aqueous phase containing the RNA was transferred to a new tube. RNA was precipitated using an equal volume of isopropanol, resuspended in RNase-free water, and stored at −80°C. Contaminating genomic DNA was removed with DNase (Promega RQ1 RNase-Free DNase, M6101) prior to analysis. For RT-PCR, cDNA was generated using the SuperScript III First-Strand System (Invitrogen, 18080051) with a primer specific for either the human *ACE2* transcript (Human *ACE2* rtB: 5′-GACTGCTTTCTGAACATTTC-3′) or the mouse Ace2 transcript (*Mus Ace2* rtB: 5′-GACAACTTTCCGGCCATCTG-3′). Human-specific PCR primers (*ACE2* BF1: 5′-CACCATTGAGGAACAGGCCA-3′ and *ACE2* BR1: 5′-TGAACATTTCCTGGGTCCGT-3′) or mouse-specific PCR primers (*Mus Ace2* BF1: 5′-CCTCACCGAGGAAAATG-3′ and *Mus Ace2* BR1: 5′-CGGCCATCTGCTGGCTCAGT-3′) were used with Choice Taq Blue DNA polymerase (Thomas Scientific, C775Y30) and the following cycling conditions: 94°C for 3 min, 32 cycles of amplification (94°C for 50 s, 54°C for 30 s, 72°C for 55 s), and 72°C for 5 min. These two assay primers bind in the first and eighth exons of the genomic sequence of the human *ACE2* gene or mouse *Ace2* gene, separated by over 19 kb in the human genome and by over 27 kb in the mouse genome, but they amplify the expected 963-bp fragment in *ACE2* or *Ace2* mRNA transcripts. For the *ACE2* and *ACE2-DT* RT-PCR assay shown in Fig. 2, the following primers were used: *ACE2* v2 rt1: 5′-TCCAAGAAGCAAGTGAACTTTGA-3′ (cDNA primer), *ACE2* v2 F1: 5′-AATGAGGACACTGAGCTCGC-3′ and *ACE2* v2 R1: 5′-GGTCTTCGGCTTCGTGGTTA-3′ (PCR primers); *ACE2*-DT rt1: 5′-AAATAGGTTGGCTTCACCACCA-3′ (cDNA primer), ACE2-DT F2: 5′-GAGGACAGCACACGAGTATCT-3′ and *ACE2-DT* R2: 5′-ATGCACTTGCTCCTCAACTCT-3′ (PCR primers).

### ACE2 protein expression and analysis of tissue pathology

Following euthanasia, lung, heart, and brain tissues were embedded in 4% paraformaldehyde for 24–48 h, rinsed in water, and stored in 70% ethanol. In some studies, lungs were inflated with 4% paraformaldehyde before isolation. Following paraffin embedding, the tissues were sectioned and stained with H&E. Stained slides were analyzed by the University of Minnesota Comparative Pathology core. For analysis of ACE2 expression, tissues were stained with anti-ACE2 Ab (MAB933, R&D Systems), and binding detected using an ARK peroxidase kit (K3954, Dako), using 3,3-diaminobenzidine (DAB) as substrate.

### SARS-CoV-2 mouse infection

For infection, we used SARS-CoV-2 strain 2019-nCoV/USA_WA1/2020 (provided by World Reference Center for Emerging Viruses and Arboviruses at the University of Texas Medical Branch). Viral stocks for mouse infection were generated by a single-passage expansion through Vero E6 cells. PFU titers were determined, and stock batch concentrations were standardized to 1 × 10^7^ PFU/ml.

Mice were anesthetized with 3% isoflurane and inoculated intranasally with 2 × 10^4^ PFU SARS-CoV-2 in a volume of 50 μl DMEM, administered equally into both nostrils (nares). Mice were monitored and weighed daily. Animals losing 25% or more of their starting weight were sacrificed, as were animals that became ataxic, paralytic, unable to right themselves, or showed labored breathing. Animal studies were conducted in an A-BSL3 facility under a protocol approved by the University of Minnesota Institutional Animal Care and Use Committee and in accordance with the Guide for the Care and Use of Laboratory Animals of the National Institutes of Health.

### SARS-CoV-2 viral load quantitation

Animals were sacrificed, and their tissues were harvested at 3, 7, or 14 d postinfection. Animals were euthanized by CO_2_ inhalation. Indicated tissues were isolated and then homogenized in M tubes (Miltenyi Biotec) with 2.5–3.0 ml RLT Plus Buffer (Qiagen) supplemented with  -ΜΕ and DX antifoaming reagent (Qiagen) and run on a GentleMACS Dissociator (Miltenyi Biotec) with the RNA 2.01 setting. Tissue homogenates were clarified by centrifugation at 10,000 rpm for 5 min and stored at −80°C. RNA was extracted from homogenized organs using the RNeasy Plus Micro Kit (Qiagen), and cDNA was generated by reverse transcription using SuperScript II (Invitrogen). RNA was normalized to the endogenous α-tubulin primer probe set: 5′-GCCTGGACCACAAGTTTGAC-3′ and 5′-TGAAATTCTGGGAGCATGAC-3′. To determine the SARS-CoV-2 genome copy number in each sample, a standard curve was generated using serial dilutions of quantitative synthetic RNA (BEI, NR- 52358) and virus-specific nsp14 primers: 5′-TGGGGYTTTACRGGTAACCT-3′ and 5′-AACRCGCTTAACAAAGCACTC-3′. The genome copy number in each sample was derived from this curve.

### Serological response to SARS-CoV-2

Mice were bled from the facial vein. Blood was clotted for at least 30 min at room temperature before centrifugation at 1500 × *g* for 15 min to fractionate serum. Serum samples were incubated for 1 h at 56°C to inactivate any SARS-CoV-2 virus present and tested in a laboratory-developed ELISA using anti-mouse reagents. The laboratory-developed ELISA was previously validated against commercially available assays and detects IgM, IgA, and IgG (26). Positive control serum was generated from mice immunized against the S receptor binding domain (S-RBD). Negative serum was from naive wild-type (WT) mice. The SARS-Cov-2 S-RBD Ag was used to coat the ELISA plates. Serial dilutions were reported at 1:50, 1:150, 1:450, 1:1,350, 1:4,050, and 1:12,150.

### Quantification and statistical analysis

Statistical significance was assigned when *p* values were <0.05, using one- or two-way ANOVA, as indicated, in Prism version 10 (GraphPad Software).

## Results

### Expression of ACE2 mRNA and protein in *ACE2*-GR mice

Because ACE2 plays an important role in homeostasis of various tissues, we sought to avoid ACE2 overexpression in mice engineered to express transgene-encoded hACE2 protein. Accordingly, our infection model uses *ACE2*-GR mice produced by deleting 71 kb of the mouse genome that encodes the mouse Ace2 protein in C57BL/6 background embryonic stem (ES) cells and subsequently inserting the 65.8-kb syntenic *ACE2* segment of the human genome to generate *ACE2*-GR mice (Fig. 1A). This approach both introduces the human *ACE2* gene locus with defined upstream and intragenic regulatory elements (20) and also eliminates the mouse *Ace2* locus, circumventing potential issues of overexpression of mouse/human ACE2, which is a concern in hACE2 transgenic models.

We confirmed the expression of human *ACE2* transcripts and elimination of mouse *Ace2* transcripts in *ACE2*-GR mice using RT-PCRs specific for either the human or mouse transcripts (Fig. 1B). For this analysis, we used total RNA isolated from kidney, a tissue in which the *ACE2* gene is highly expressed (21). As expected, we detected expression of only mouse *Ace2* transcripts in WT mice with the endogenous mouse *Ace2* allele, only human *ACE2* transcripts in mice homozygous for the *ACE2-GR* allele, and both transcripts in mice heterozygous for the *ACE2*-GR and mouse *Ace2* alleles (Fig. 1B). Further analysis determined whether some of the reported alternative transcripts in the *ACE2* locus (20, 24, 25, 27) could be detected in the *ACE2*-GR mice (representative results are shown in Fig. 2). All the transcripts tested (including the long noncoding RNA transcript *ACE2-DT* [27]) were detected in the kidney, whereas expression in other tissue sites varied, as expected (20, 21). These data indicate that *ACE2*-GR mice provide a model to investigate the significance of distinct *ACE2* transcripts in its biological function and as a viral entry target.

**FIGURE 3.**
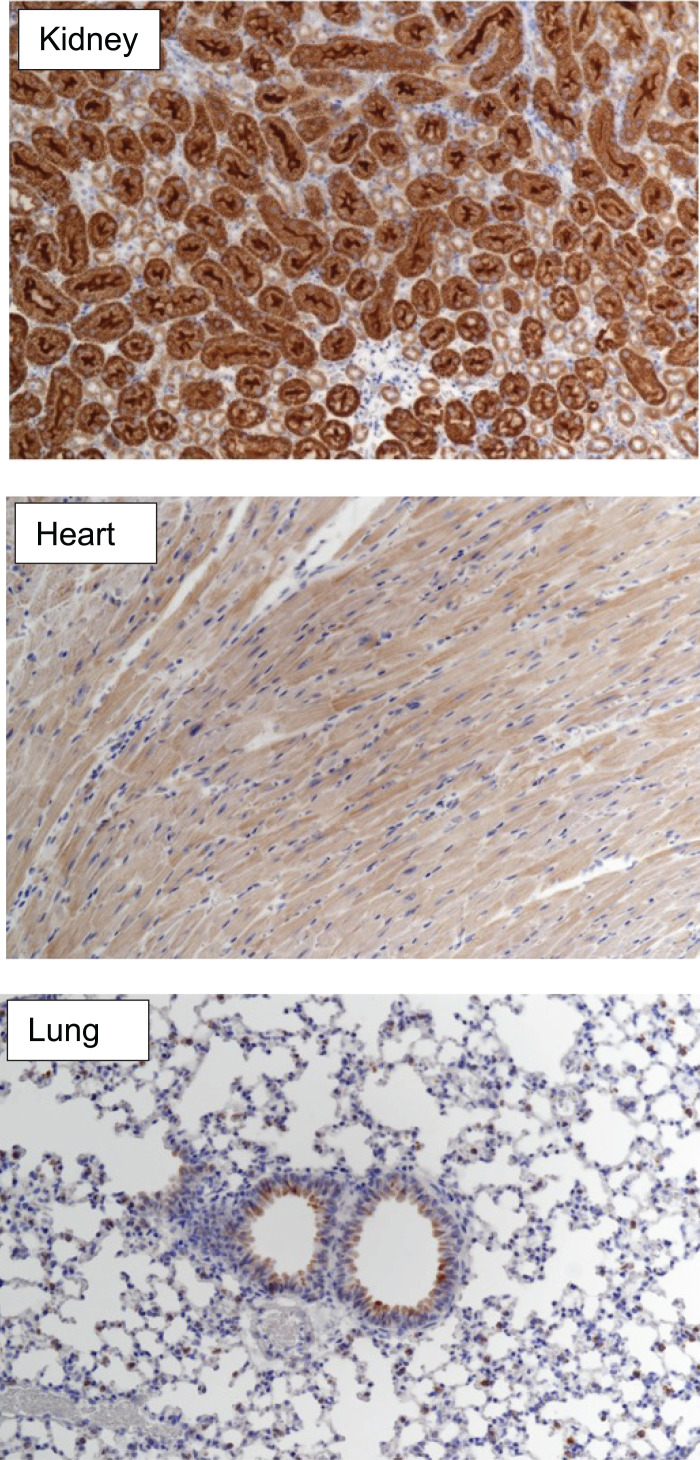
In situ human ACE2 protein expression in *ACE2*-GR mice. ACE2 expression (brown staining) in tissue sections from the kidney (top), heart (middle), and lung (bottom) of *ACE2*-GR mice. Because these homozygous *ACE2*-GR mice lack the mouse *Ace2* gene, protein expression derives exclusively from the human *ACE2*-GR locus. Sections were counterstained with hematoxylin (blue) (original magnification ×20).

To evaluate tissue- and cell-specific expression of the human ACE2 protein, we performed immunohistochemistry of tissues from *ACE2*-GR mice (Fig. 3). We found that human ACE2 is strongly expressed in the kidney tubules, detectable in some epithelial cells of the lung and diffusely expressed in heart muscle, mirroring the expression pattern of mouse Ace2 in normal mice and human ACE2 expression in humans (21), indicating that the regulatory elements introduced by gene replacement faithfully recapitulate tissue-specific expression of human ACE2.

### Assessing susceptibility of *ACE2*-GR mice to SARS-CoV-2 infection

**FIGURE 4.**
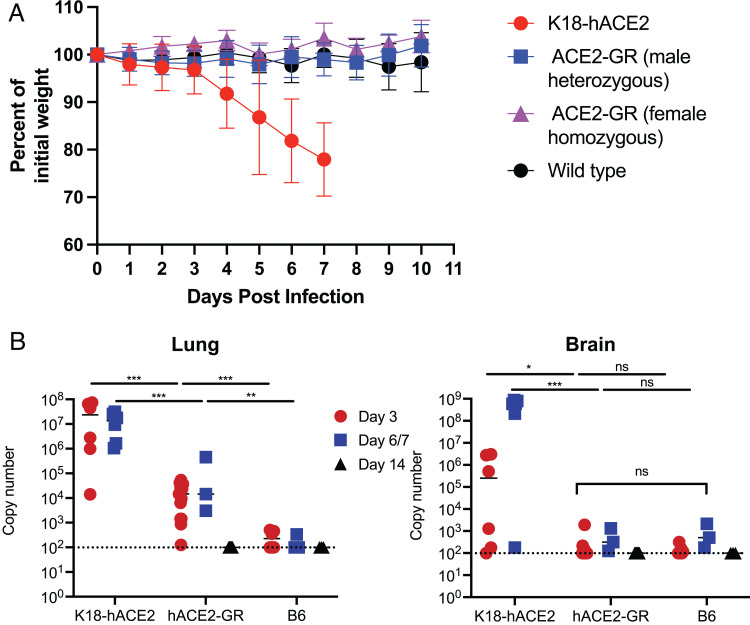
*ACE2*-GR mice survive infection with SARS-CoV-2 and support viral replication in the lungs but not the brain. (**A**) Weight loss of B6 WT, K18-hACE2, and *ACE2*-GR mice intranasally infected with SARS-CoV-2 (WA). (**B**) SARS-CoV-2 viral load in lungs (left) and brain (right) tissues isolated at the indicated time points. Viral loads could not be determined at day 14 in K18-hACE2 mice, because these animals had to be sacrificed by day 7. Data in (A) are from 6–10 mice per group and representative of three experiments. Data in (B) are from —five to eight animals per group and are representative of three independent experiments. Statistical analysis (one-way ANOVA) is represented as *p < 0.02; **p < 0.005; ***p < 0.001.

Our objective with generation of *ACE2*-GR mice was to produce a mouse model that would permit infection by SARS-CoV-2 but avoid the fatal immunopathology induced in other mouse models, in which transgenic ACE2 is ectopically expressed. For example, the widely used K18-hACE2 transgenic strain expresses ACE2 strongly in the various epithelia, and infection by SARS-CoV-2 (or by SARS-CoV-1) induces death, associated with viral replication that extends to the CNS (15, 17). Indeed, intranasal infection of K18-hACE2 mice with SARS-CoV-2 (2 × 10^4^ PFU USA-WA1/2020 strain) caused rapid weight loss, necessitating sacrifice of the animals by day 7 postinfection (Fig. 4A). This was associated with sustained high viral loads in the lungs and dramatically increasing viral load in the brains of infected K18-hACE2 mice (Fig. 4B) during the week following SARS-CoV-2 infection, similar to previous reports (17, 18). WT C57BL/6 mice showed no weight loss and minimal evidence for SARS-CoV-2 viral replication in either the lungs or the brain (Fig. 4A, 4B). In contrast, although *ACE2*-GR mice showed no detectable weight loss following SARS-CoV-2 infection, they nevertheless showed clear evidence for viral replication in the lungs but not in the brain at both days 3 and 6/7 following SARS-CoV-2 infection (Fig. 4A, 4B). By day 14 postinfection, SARS-CoV-2 could not be detected above background in *ACE2*-GR mice. Hence, our data indicate a stark difference in the SARS-CoV-2 viral load and tissue distribution in K18-hACE2 and *ACE2*-GR mouse strains, the latter providing a model in which SARS-CoV-2 replication is sustained for at least 1 wk in the lungs of infected mice but does not spread to the CNS.

Because both mouse *Ace2* and human *ACE2* are encoded on X chromosomes, it was of interest to determine whether there was a difference between male and female *ACE2*-GR mice in their vulnerability to SARS-CoV-2 infection; however, we observed no substantial differences in weight loss or viral load related to the sex of the *ACE2*-GR mice (Fig. 4A and data not shown).

**FIGURE 5.**
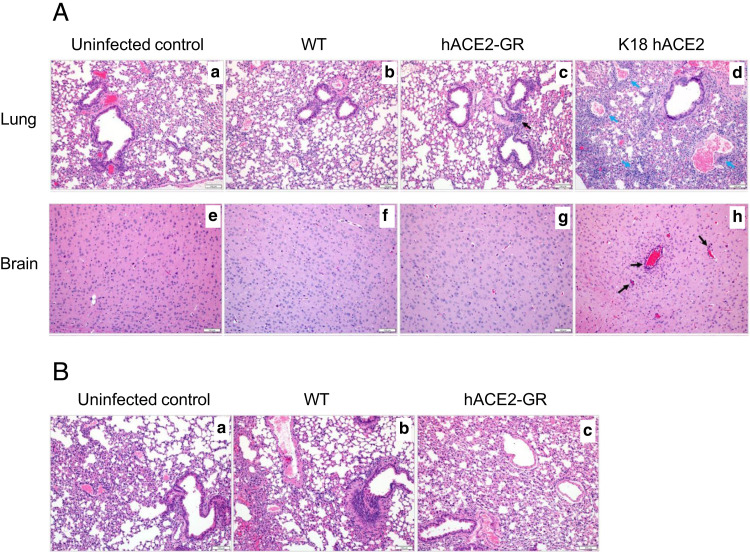
Lung and brain immunopathology following infection with SARS-CoV-2 in mouse models. Histopathology of formalin-fixed, paraffin-embedded tissues stained with H&E. (**A**) Lungs (**a–d**) and brains (**e–h**) from uninfected WT control animals (a, e) and from WT (b, f), *ACE2*-GR (c, g), and K18-hACE2 transgenic mice (d, h), 6 d after SARS-CoV-2 infection. Black arrows in (c) indicate foci of peribronchial lymphoid tissues; blue arrows in (d) mark perivascular inflammatory infiltrates; and black arrows in (h) identify sites of mononuclear perivascular meningoencephalitis. (**B**) Lungs from uninfected WT mice (**a**) and from WT (**b**) and *ACE2*-GR (**c**) mice 14 d after SARS-CoV-2 infection. Infected K18-ACE2 transgenic mice did not survive to day 14. All images are at 100× original magnification and are representative of two experiments with four or five mice per group.

To assess tissue pathology associated with intranasal SARS-CoV-2 infection, we conducted histological analysis of lung and brain tissues from WT, K18-hACE2, and *ACE2*-GR mice (Fig. 5). At 6 d postinfection, WT mice showed no significant signs of lung or brain inflammation, whereas *ACE2*-GR animals showed occasional peribronchial lymphoid aggregates, and neither group showed infiltrates in the brain (Fig. 5A). In contrast, K18-hACE2 transgenic mice had marked mononuclear infiltrates in the lung and the brain (Fig. 5A). By day 14 postinfection, infiltrates in the lung had resolved in the WT and *ACE2*-GR mice (Fig. 5B). K18-hACE2 mice could not be analyzed at this time point, because they had died of the infection.

### ACE2-GR mice make an Ab response to the SARS-CoV-2 S protein

**FIGURE 6.**
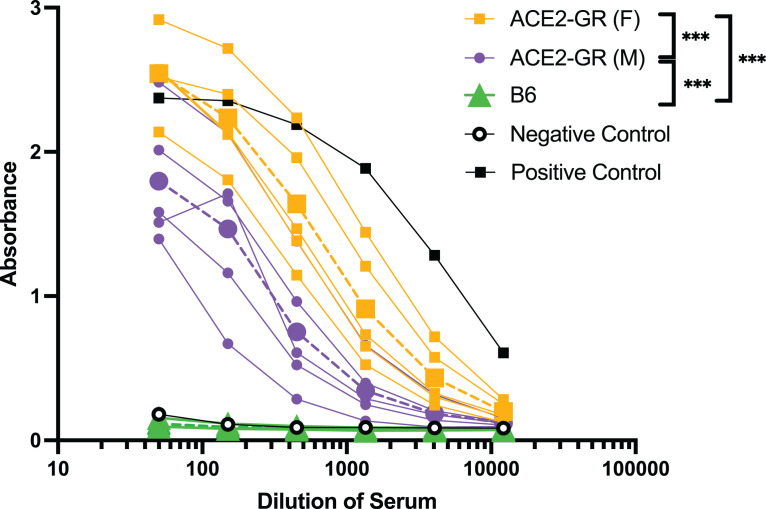
Consistent serological responses to SARS-CoV-2 in *ACE2*-GR mice. B6 and *ACE2*-GR mice were intranasally infected with SARS-CoV-2 (WA) and Ab titers against the SARS-CoV-2 S-RBD were measured by ELISA (measuring all isotypes). Average titers of each group (*n* = 3–5) are shown with dotted lines. Statistical significance was determined by two-way ANOVA analysis of average values: ***p < 0.0001. Positive control samples were from mice vaccinated against S-RBD, whereas the negative control serum was from naive WT mice. Data are representative of two independent experiments.

Finally, we conducted an initial assessment of the immune response to SARS-CoV-2 in *ACE2*-GR mice through analysis of the serological response to the RBD of the SARS-CoV-2 S protein 14 d following infection. SARS-CoV-2–infected *ACE2*-GR mice consistently mounted a strong Ab response (Fig. 6). In contrast, there was typically no detectable serological response in infected WT mice (Fig. 6), although in some individual experiments, a strong response was detected, presumably reflecting immune reactivity to the viral inoculum (data not shown). Interestingly, we observed that the serological response was of a greater magnitude in female than in male *ACE2*-GR mice (Fig. 6).

## Discussion

Since the COVID-19 pandemic began, numerous animal models have been established to study SARS-CoV-2 infection, the pathology it induces, and the nature of protective immune responses against the virus. At the outbreak of the pandemic, when our studies began, the K18-hACE2 model was widely used. This model, developed following the SARS-CoV-1 outbreak, permits effective intranasal infection through hACE2 by either SARS-CoV-1 or SARS-CoV-2. However, this model suffers from ectopic expression of hACE2 and spread of the virus to the CNS, which correlates with a typically fatal progression of the disease within 1 wk of the infection (17, 18). Although valuable as a model to assess the protective effects of preexisting immunity, the rapid demise of K18-hACE2 mice limits the ability to track induction of the primary immune response. Furthermore, it is not clear whether the CNS pathology observed in SARS-CoV-2–infected K18-hACE2 mice reflects a course of viral spread that ever arises in immunocompetent humans. The fatal disease observed in the K18-hACE2 model also precludes its adoption for studies on nonlethal SARS-CoV-2 infections, such as those that lead to successful resolution of the infection or the induction of “long COVID.” Nevertheless, models such as the K18-ACE2 transgenic strain fulfill a valuable role for studying induction of lethal disease following SARS-CoV-2 infection and the efficacy of vaccines or therapeutics to prevent pathology.

At least in part, limitations of K18-hACE2 mice as a model for human SARS-CoV-2–induced disease likely arise because of the dysregulated expression pattern of the hACE2 transgene. Although expression of the full-length *ACE2* cDNA under the K18 promoter effectively allows transgene expression in various epithelia, including those in the lung, the regulation of gene expression does not match what is observed in humans. Intriguingly, despite the justifiable focus on human ACE2 protein expression in lung cells as the route for SARS-CoV-2–induced lung pathology, ACE2 expression is extremely low in the human lung compared with the kidney, for example (21). Other transgene ACE2-expressing mouse models, generated following the start of the COVID-19 pandemic, suffer from some of the same limitations. Transient expression of hACE2 induced by intranasal infection with recombinant adenovirus or adeno-associated virus models allows focal SARS-CoV-2 infections in the lung, but these cannot be sustained because of the loss of transduced cells (16). Other transgenic models have been generated, including mice expressing human *ACE2* cDNA under control of the mouse *Ace2* regulatory elements (18) and, more recently, animals with low copy numbers of the K18-ACE2 transgene (28). Although valuable, these models are necessarily confined to expression of one full-length *ACE2* transcript, whereas it is known that humans express various alternative spliced forms of *ACE2* (some of which are unable to bind the SARS-CoV-2 S protein) (20–24).

A report published after deposition of our *ACE2*-GR model at The Jackson Laboratory (in August 2021) used a similar approach of syntenic locus replacement (20). Snouwaert et al. reported similar findings on human ACE2 expression and transient SARS-CoV-2 infection in their humanized mouse models, but it is worth noting that the *ACE*-GR strain characterized in the present study involves a considerably larger region on the human *ACE2* promoter, including the full sequences of the putative long noncoding RNA, *ACE2*-DT (27), which is not present in the ACE2 humanized mice described by Snouwaert and colleagues (20). Furthermore, those ACE2 humanized lines were generated using 129SvEv ES cells, whereas we used C57BL/6J ES cells, allowing compatibility with the abundant transgenic and gene knockout models on the C57BL/6 genetic background.

The more physiologically regulated expression of human ACE2 protein in *ACE2*-GR mice will offer an improved model to assess how infection with SARS-CoV-2 strains and subsequent immune responses are affected by inflammatory diseases, immunodeficiencies, and aging. For this initial report, we focused on the response to the USA-WA1/2020 strain; further studies will be needed to compare the response of ACE2-GR mice to infection with SARS-CoV-2 variants. ACE2 expression and alternative splicing are found to increase in response to inflammatory cues, including IFNs, potentially influencing SARS-CoV-2 infection (5, 20). The low expression of ACE2 in lung cells of *ACE2*-GR mice presents an opportunity to investigate this dynamic in a tractable model. Our studies suggested potential sex differences in the serological response to SARS-CoV-2 infection of *ACE2*-GR. Although the basis for this finding is not clear, it will be interesting to investigate further in light of sex differences in COVID-19 susceptibility.

Nevertheless, the *ACE2*-GR model has limitations in modeling SARS-CoV-2 infection, because it contains only a single human gene (*ACE2*). The other host genes involved in viral infection and replication and in the immune response to this infection are all still those of mice, and some of their functions are likely to differ significantly from those of a human host. Furthermore, the model assumes that mouse factors regulating chromatin accessibility and gene expression will act on the human *ACE2* locus to provide a “normal” expression pattern.

In summary, the *ACE2*-GR infection model described in the present study used human ACE2 protein expressed from the full *ACE2* gene in its normal genomic context, offering new opportunities to apply this mouse model to studies on SARS-CoV-2 and related coronaviruses.
